# Laser Ablation Sampling With Low‐Power Plasma: A LA‐MIP‐MS Instrument for Spaceflight

**DOI:** 10.1002/rcm.10152

**Published:** 2025-11-24

**Authors:** Benjamin J. Farcy, Jacob Graham, Madeline Raith, Ricardo Arevalo, Mazdak Taghioskoui, Sierra Budinoff, Amy McAdam, Jane Lee, Ryan M. Danell, Desmond A. Kaplan, Cynthia Gundersen, William F. McDonough

**Affiliations:** ^1^ Department of Astronomy University of Maryland College Park Maryland USA; ^2^ NASA Goddard Space Flight Center Greenbelt Maryland USA; ^3^ Department of Geology University of Maryland College Park Maryland USA; ^4^ Trace Matters Scientific LLC Bethesda Maryland USA; ^5^ AMU Engineering Miami Florida USA; ^6^ Danell Consulting Inc. Winterville North Carolina USA; ^7^ KapScience LLC. Tewksbury Massachusetts USA; ^8^ Tohoku University Advanced Institute for Marine Ecosystem Change, and Department of Earth Science Sendai Miyagi Japan

**Keywords:** geochemistry, laser ablation, mass spectrometry, plasma, spaceflight

## Abstract

**Rationale:**

Inductively coupled plasma (ICP) is a commonly used ion source for mass spectrometry‐based chemical analysis of a wide range of materials. Traditional ICP ion sources use high power (> 1000 W) and significant gas flow (> 10 L/min), rendering them unsuitable for spaceflight, as they are too resource‐intensive for planetary spacecraft.

**Methods:**

To address the technology gap, we designed and developed a laser ablation microwave‐induced plasma mass spectrometer (LA‐MIP‐MS) and experimentally validated the analytical performance of a prototype instrument capable of providing in situ analyses during planetary science missions. We developed a low‐pressure plasma ion source and interfaced it to a heritage quadrupole mass spectrometer (QMS) to perform elemental and isotopic analysis of solid samples via laser ablation. The low power plasma ion source was generated at < 1 Torr (< 133 Pa) using 30 W of power and 50 mL/min of He. Analytes were introduced via laser ablation (266 nm); we report elemental abundances and isotopic ratios for Cu, Ni, and Fe metals.

**Results:**

Our experiments confirmed quantification accuracy for stainless steel within 1.4–4% of values measured by x‐ray fluorescence (XRF), with precision ranging from ±9.1 to 22% (2σ_m_). Cu and Ni isotopic ratios were measured with ±0.8–3% (2σ_m_) precision and reproducibility ranging from 0.12% to 11.8%. Measured limits of detection ranged from 21 ppmw for ^57^Fe to 780 ppmw for ^54^Fe, with limits of detection for Cr, Mn, and Ni below 240 ppmw.

**Conclusions:**

This technique adds to the roster of instrumentation available for planetary missions by enabling elemental and isotopic analysis with orders of magnitude less power and plasma gas relative to commercial ICP‐MS systems. This work paves the way for low resource LA‐MIP‐MS instruments as a viable technique to be applied to a wide range of applications for terrestrial and spaceflight chemical analysis of geologic materials.

## 1 Introduction

The past five decades have heralded an unprecedented era of planetary exploration, employing landers, rovers, and orbiters to surfaces and orbits of planetary bodies throughout the solar system. These investigations included numerous scientific instruments for in situ analysis of the chemistry of planetary surfaces and atmospheres. Mass spectrometers, in particular, with a range of ion sources and analyzers, have provided onsite compositional analyses of the exospheres or atmospheres of the Moon [1, 2, 3], Mars [4, 5, 6], Saturn [7, 8, 9], Jupiter [10], Titan [11], and Venus [12, 13], as well as Martian rocks and soils [14, 15, 16, 17]. These instruments have provided valuable chemical context to landed planetary missions, including age constraints on Martian sediments [18, 19]. A variety of mass spectrometer types, including sector field (e.g., Apollo, Viking), quadrupole (e.g., Pioneer Venus, Cassini‐Huygens, Mars Science Laboratory (MSL), Lunar Atmospheric and Dust Environment Explorer (LADEE), Mars Atmosphere and Volatile EvolutioN (MAVEN)), linear ion trap (e.g., ExoMars, Dragonfly), and time‐of‐flight (e.g., Rosetta) have been sent on previous missions or will be sent on future missions. The various mass spectrometer types are fitted with ion sources, where almost all previous spaceflight instruments have leveraged an open or closed ion source, relying on electron ionization (EI) filaments to ionize neutral species of interest. However, EI sources are limited to the analysis of gases and volatile organic species, precluding the analysis of elemental analytes from geologic material.

For in situ quantification of major, minor, and trace elements in planetary surface materials, state‐of‐the‐art measurement techniques currently rely on spectroscopy, such as laser‐induced breakdown spectroscopy (LIBS), a technique pioneered for spaceflight on MSL [20, 21] and Perseverance rovers [22], and alpha particle x‐ray spectroscopy (APXS), a method used in the Mars Pathfinder [23], Mars Exploration Rover (MER) [24], and MSL mission [25, 26]. However, spectroscopy‐based measurement methods have fundamental limitations, such as the inability to measure all elements in the Periodic Table, much less their isotopic ratios. Several mass spectrometry systems have been employed to perform in situ analyses; for example, pyrolysis of evolved gas by the MSL/SAM instrument was performed on Martian soil and rock samples. However, this technique only constrains sample materials that evolve volatiles on heating (e.g., clay minerals, sulfate salts, carbonates) [17, 27]. Thus, in order to expand the capabilities for geochemistry on planetary surfaces via mass spectrometry, new instrumentation for geologic sampling is needed.

In terrestrial laboratories, inductively coupled plasma mass spectrometry (ICP‐MS) is the gold standard for geochemistry measurements. ICP‐MS uses a high temperature radio‐frequency (RF) plasma to atomize and ionize sample material, enabling elemental and isotopic analyses of nearly all elements on the Periodic Table. The plasma is maintained using a typical Fassel torch design, in which an outer sheath of gas contains and restricts an inner plasma gas, which can reach temperatures as high as 10,000 K [28]. The sample is injected into the center of this plasma via a He injector gas and is then ionized and introduced into the mass spectrometer. This design, in conjunction with sampling/skimmer cone front‐end and a quadrupole mass analyzer, has enabled limits of quantifications as low as parts‐per‐trillion levels for elemental species, as well as isotope ratios with uncertainties in the parts‐per‐ten thousand level, establishing ICP‐MS as the standard for analyzing low‐abundance species in geologic material. However, the plasma torch requires power inputs of > 1000 W, and gas flow rates of > 10 L/min of Ar and/or He, rendering the conventional architecture too resource‐intensive for spaceflight. Spaceflight instruments have strict limits on power requirements, with most systems not exceeding a few hundred watts in total power consumption. Furthermore, high gas flow would require prohibitively large amounts of plasma gas included in the instrument's design. Therefore, these restrictions require significant modifications to the plasma ion sources to make them compatible for planetary exploration.

Previous work has been carried out to reduce the power and gas flow of atmospheric pressure ICP torches for chemical analysis. Attempts to redesign the torch with conical, spherical, or annular geometries, as well as the implementation of water‐cooled and air‐cooled torch designs, have resulted in reduced gas flow consumption as low as 1.5 L/min, with reduced power input ranging from 650 to 900 W [29, 30, 31]. Other approaches have used direct current (DC) discharge and glow discharge tubes to generate plasma, known as atmospheric pressure ionization (API) plasmas, and have been interfaced to commercial mass spectrometers and demonstrated detection and quantification of elemental and organic analytes [32, 33, 34]. Similar efforts to reduce plasma power and gas consumption with plasma ionization included low temperature discharge for ambient desorption and ionization via dielectric barrier discharge [35, 36], electrospray‐based plasma ionization [37], and ambient ionization via He‐based plasma discharge. Each technique required < 3 to 13 W of power as DC discharge sources, and 1–2 L/min of gas flow to maintain the plasma and transport sample.

Microwave frequency plasmas (e.g., > 2 GHz) have also been shown to produce reliable plasma ionization for chemical analysis. Microwave induced plasmas (MIPs) are known for lower beam dispersion due to better RF coupling to the plasma, making MIP‐generated ion sources more efficient for sample processing than lower frequency RF‐generated plasma ion sources [38, 39, 40]. Microwave cavities were first produced for plasma ionization by Fehsenfeld et al. [41] and subsequently used for spectroscopy purposes before being interfaced with commercial mass spectrometers. Previous efforts to measure elemental analytes via MIP‐MS have shown good sensitivities, although high air background and less efficient sample entrainment made the elemental sensitivity lower than typical Ar‐based ICP‐MS [42, 43].

Reducing the pressure of the plasma appears to increase the atomization and ionization efficiency for input solid laser ablation samples. In particular, low‐pressure helium plasmas have better atomization characteristics compared to atmospheric pressure argon plasmas [44]. Using a low‐pressure MIP reduced the gas and power consumption to as low as 15 W and 3 mL/min of He flow for the analysis of organic material, but a higher power was required for elemental analysis based on previous work [45, 46]. Taghioskoui and Zaghloul [47] used a low‐pressure CO_2_ plasma as an ion source for CH_4_ gas, producing molecular fragmentation with as little as 3.5 W and demonstrating the potential for atomization with low‐pressure plasmas. Farcy et al. [48] performed a Langmuir probe analysis on a He‐MIP ion source and demonstrated the ability to ionize nearly any element in the Periodic Table with only < 23 W of plasma power and < 200 mL/min of gas flow. A similar low‐pressure He‐MIP was later integrated into a ThermoNeptune multicollector mass spectrometer and demonstrated high precision analysis of gaseous oxygen isotopes [49]. Thus, low‐pressure MIP ion sources have shown promise for their analytical performance in mass spectrometry.

For commercial ICP‐MS systems, one of the most common methods of sample introduction is laser ablation (LA). LA‐ICP‐MS serves as a crucial tool in geochemistry, as it requires almost negligible sample preparation and can deliver spot precision sampling down to the tens of μm scale ([50] and references therein). However, the sample introduction system used for nearly all low‐power plasma mass spectrometry has relied on either liquid sample introduction or gas‐phase sampling to date. Low power plasmas may be challenged to process input LA sample material, as the plasma temperatures may be too low to atomize solid material or bring elemental analytes above their vaporization temperature. LA sampling is a valued sample introduction technique for spaceflight instrumentation and is currently being implemented for a number of selected missions [51, 52, 53] and advanced instrument concepts [54, 55, 56]. Thus far, LA sample introduction has not yet been demonstrated with low power, low gas flow MIP ion sources for mass spectrometry.

To evaluate the feasibility of a microwave‐induced plasma (MIP) as an ion source for chemical analysis, we developed a sub‐atmospheric helium MIP coupled to a laser ablation sample cell. We designed reduced‐pressure sampling and ion optics and implemented a quadrupole mass analyzer based on heritage hardware. Solid samples were analyzed to demonstrate proof of principle. Here, we present the design, construction, and performance of a resource‐efficient LA‐MIP‐MS prototype system, combining a low‐power, low‐gas‐flow MIP with a quadrupole analyzer derived from the Sample Analysis at Mars (SAM) instrument on the MSL Curiosity rover [27]. We demonstrate quantification of elemental abundances and isotopic ratios with precision sufficient to meet planetary science objectives, while significantly reducing resource demands for lunar and Martian mission concepts.

### 1.1 Instrument Design and Layout

**FIGURE 1:**
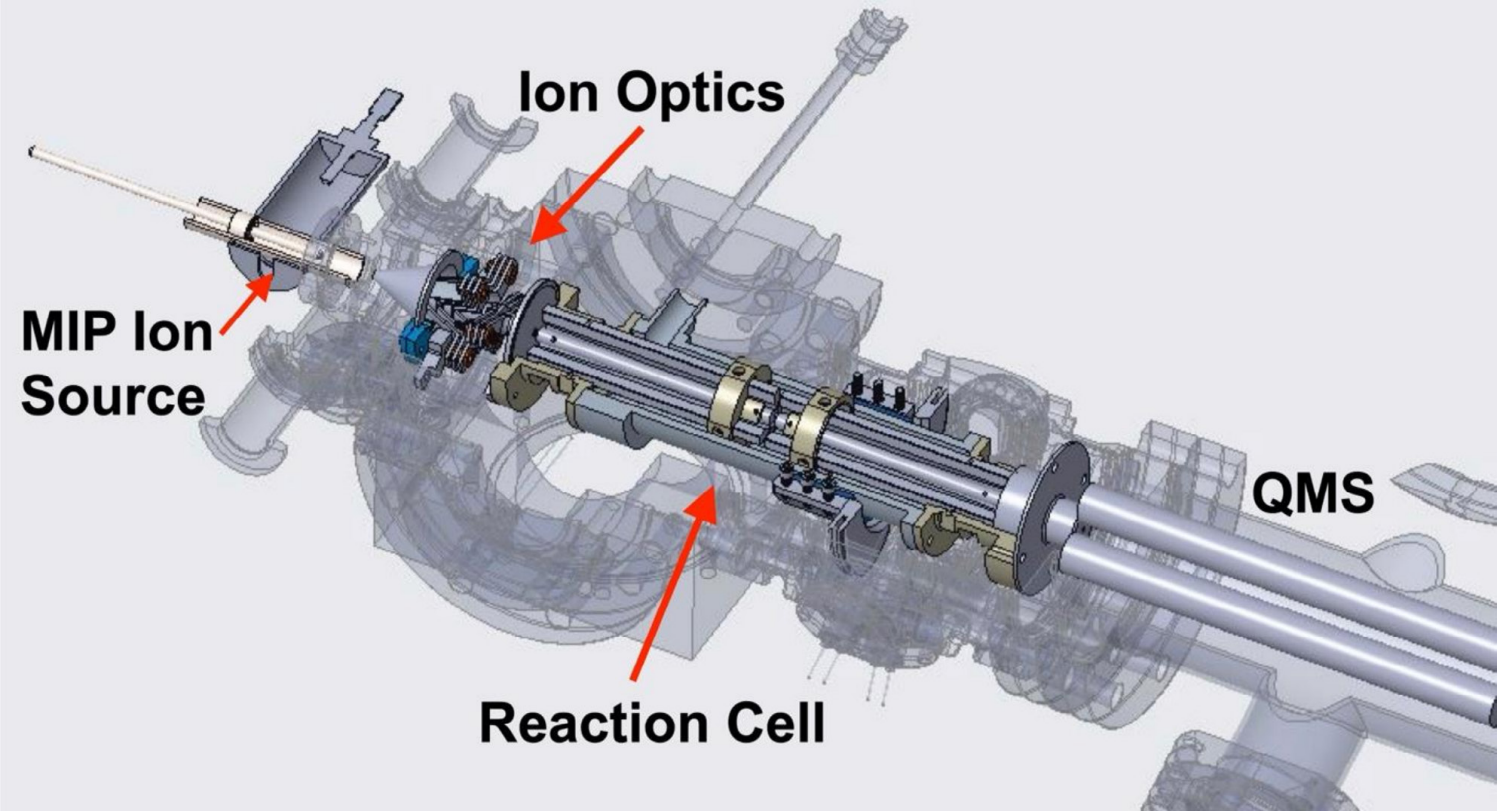
Computer‐aided design (CAD) model cross section of the MIP‐MS prototype constructed for this study, including the MIP torch and ion source, ion optics, reaction cell, and quadrupole mass spectrometer (QMS).

**FIGURE 2:**
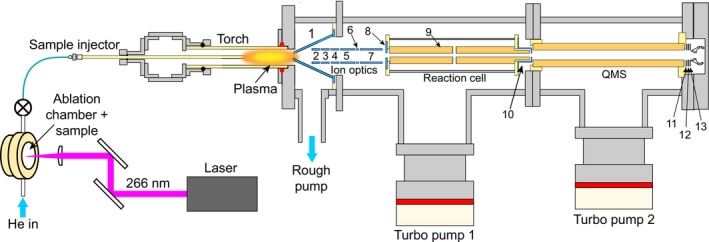
Schematic of LA‐MIP‐MS constructed for this study. Table 1 provides typical data for lens voltages, forward power, gas type and flow rates, and laser parameters.

A working prototype of the LA‐MIP‐MS instrument was constructed using spaceflight heritage hardware, integrated together with custom‐built and commercial components (Figure 1). The design of the system was informed by a breadboard version of the instrument originally developed through the NASA ROSES PICASSO program. The instrument follows the typical layout of a quadrupole ICP‐MS system, including a plasma torch, ion optics, radio‐frequency (RF) ion guide reaction cell, quadrupole mass spectrometer (QMS), and channeltron electron multiplier (CEM) (Figure 2).

### 1.2 Torch

The torch is designed to maintain an Ar or He plasma at sub‐atmospheric pressure, typically < 1 Torr (< 130 Pa), depending on gas flow rate. The torch is an alumina ceramic tube, with a ½″ (12.7 mm) outer diameter (OD) and a 3/8″ (9.5 mm) inner diameter (ID) and length of ~6″ (150 mm). Alumina, versus quartz, is used because of its low thermal conductivity, high maximum temperature, and higher mechanical strength. The torch is interfaced to the mass spectrometer via a 1 1/3″ (33.8 mm) conflat (CF) flange with a ½″ (12.7 mm) central bore and is sealed to a custom interface via a Viton O‐ring (high‐temperature formulation) compressed by the flange. The opposite end of the tube is held in place using a CF quick‐connect mounted onto a 1 1/3″ (33. mm) CF cube, ensuring that both sides are sealed to the vacuum system. To maintain low pressure inside the torch, the plasma gas and entrained ablation particles enter into the torch region through an alumina ceramic injection tube with an OD of 1/8″ (3.18 mm) and ID of 1/16″ (1.59 mm). The injection tube is held in place using a CF quick‐connect also mounted to the torch‐mounted CF cube, ensuring that the torch and sampler tube are concentric. The injection tube is interfaced to a gas transfer line, which transports the plasma gas and laser ablation particles.

The torch operates at sub‐atmospheric pressure to enable a high vacuum in the QMS portion of the instrument. To ensure that the torch environment remains at low pressure, a roughing pump (3 m^3^/h pumping speed) is continuously operated. The plasma ion source is integrated with the instrument ion optics via a single sampler cone with a 0.02″ (0.5 mm) aperture to enable differential pumping between the ion optics and the plasma torch. This torch interface setup differs from commercial systems which use a double cone interface to bring the atmospheric pressure ions into the vacuum; here, only a single cone configuration is needed due to the low‐pressure plasma torch environment. The base pressure of the torch is typically 3 × 10^−3^ Torr (0.4 Pa) without plasma gas but increases to 0.1–1 Torr (10–130 Pa) with 10–100 mL/min of He flow. A plasma gas of He is preferred over Ar due to its enhanced ionization efficiency at a given forward power [48]; the gas flow is controlled using a mass flow controller (Alicat Scientific Inc., MC‐500SCCM‐D‐DB9‐PCV10/CM).

The plasma is generated and maintained using an Evensen microwave discharge cavity (Opthos Inc. EC‐1), which resonantly transfers power from the microwave source to the gas encased in the torch [41]. The cylindrical cavity operates by increasing the electric field inside the gas environment and is tuned to minimize reflected power. The structure of the microwave cavity allows for resonance to 2.45 GHz RF, as the volume of the cavity determines the resonant frequency. The cavity can be tuned to match the input frequency via a tuning knob at the top of the cylinder, which alters the volume and resonant frequency. Tuning can also be achieved through impedance matching, which adjusts the coupling of the RF load cable to the cavity. The RF power is supplied by an RF signal generator (MiniCircuits ISC‐2425‐25+), with the signal being fed into a high‐power RF amplifier (MiniCircuits ZHL‐2425‐250X); the plasma is ignited by first flowing Ar into the torch, then using a direct current (DC) spark discharge inside the plasma torch. The Ar flow is used for plasma ignition due to its lower required voltage to produce a spark discharge. Once plasma ignition occurs, the gas is changed from Ar to He and the cavity is tuned using the resonant tuning knob and the impedance matching stub to minimize reflected power.

### 1.3 Ion Optics

A custom set of ion optics was designed, fabricated, and mounted onto a custom‐built flange that also supports the sampler cone, pumping port, and torch interface. The ion optics define the electric fields that influence ion trajectories after leaving the torch, ultimately shaping and focusing the beam upon entry into the mass analyzer. DC voltages are supplied to each of the ion optic lens elements with a multi‐channel, programmable voltage source (GAA Custom Electronics LLC), and controlled via in‐house software [57]. Ions entering the first stage of the instrument from the torch through the sampler cone are extracted at a negative voltage (Table 1). This differs from commercial ICP‐MS systems, in which the inlet sampler cone is typically grounded to remove plasma electrons and maximize positive ion extraction throughput. The beam shaping ion optics include three lenses that collimate and accelerate the ion beam, followed by an Einzel lens stack to focus the beam into the reaction cell inlet. The ion optics region has a base pressure of 2 × 10^−6^ Torr (3 × 10^−4^ Pa) that increases to 2 × 10^−4^ Torr (3 × 10^−2^ Pa) when plasma gas flows.

**TABLE 1:** Laser and MIP‐MS operating parameters.

Laser	
Beam shape	Elliptical
Spot size (minor axis)	50 μm
Spot size (major axis)	100 μm
Energy output (266 nm)	500 μJ
Pulse duration	9 ns
**MIP**	
Forward power	30 W
Reflected power	<200 mW
Gas type	He
Gas flow rate	30–50 mL/min
**Ion optics**	
Sampler cone (1)	‐ 15 V
Focus 1 (2)	−190 V
Focus 2 (3)	−50 V
Focus 3 (4)	−120 V
Einzel 1 (5)	−150 V
Einzel 2 (6)	20 V
Einzel 3 (7)	−165 V
Reaction cell entrance plate (8)	−55 V
Reaction cell front rod bias (9)	−120 V
Reaction cell rear rod bias (10)	−140 V
Reaction cell exit lens (11)	−230 V
Reaction cell RF frequency	2.14 MHz
Reaction cell RF voltage (V_pp_)	120 V
QMS bias	4 V
Detector focus 1 (12)	−32 V
Detector focus 2 (13)	−192 V
Detector focus 3 (14)	45 V
CEM detector bias	−2500 V

### 1.4 Reaction Cell

An RF multipole ion guide efficiently transports ions between the ion optics and the QMS. The ion guide subsystem is enclosed by a sealed housing that includes a gas inlet line, allowing sample ions to react with a selected background gas (e.g., N_2_O) to separate isobaric interferences (e.g., ^87^Rb‐^87^Sr). Although this capability is not demonstrated here, the separation of ^87^Rb and ^87^Sr using a similar reaction cell design has been described elsewhere [58, 59]. The reaction cell subsystem was extracted from a commercial triple‐quad ICP‐MS (SciEx Inc.) instrument and modified to interface with the heritage QMS by extending the rods of the multipole and adding a custom end‐cap electrode for efficient extraction of ions. The reaction cell rods are driven using the same multi‐channel voltage source that supplies the DC voltages. This unit controls a resonant RF power supply and can measure its performance and drive its operation frequency. For this setup, the output is typically 2.14 MHz with a controlled amplitude based on the mass range being investigated. An amplitude of 20–30 V peak‐to‐peak is used to transmit low mass ions (*m/z* ≤ 40), while 120 V peak‐to‐peak is applied for higher mass ions (*m/z* > 80).

### 1.5 Quadrupole Mass Spectrometer

Ions exiting the reaction cell are filtered by their *m/z* ratio using a scanning QMS with spaceflight heritage. The QMS is based on the robust design of the SAM instrument; similar core designs have been flown on multiple planetary missions (e.g., Cassini‐Huygens, MAVEN, and LADEE). The QMS uses hyperbolic rods, but unlike the SAM instrument, it only uses a single RF frequency of 2.1 MHz, controlled by a commercial quadrupole power supply (Extrel QPS2000). The QMS is integrated with a Photonis 4870 CEM capable of a gain of up to 5x10^8^ at maximum voltage (i.e., 3000 V), with the ion signal recorded in pulse counting mode using a commercial preamp and count‐rate to analog converter (Advance Research Instruments Corporation).

### 1.6 Laser Ablation Cell

A custom‐designed laser ablation cell was fabricated and integrated into the plasma torch. Unlike laser ablation cells used in commercial LA‐ICP‐MS systems, this cell is evacuated through the sample transfer line to the sub‐atmospheric pressure of the torch. Given the focus on quantifying elemental abundances and isotopic ratios (versus the organic composition of the sample), the prototype ablation cell was 3D printed and made with Vero, a rigid, general purpose polymer resin capable of maintaining vacuum pressures. The cell is designed to hold standard 1″ (25.4 mm) round samples and is equipped with a UV transmissive CaF window to facilitate the transmission of incident UV laser light (266 nm, 9‐ns pulse duration) for sample ablation. The cap and body of the cell, as well as the transmissive window, are sealed with Viton O‐rings to maintain a vacuum seal.

He gas flows across the sample surface, entrains ablation particles, and exits the cell into the gas transfer line. A PEEK capillary tube with ID 0.028″ (0.7 mm) is interfaced to the exit of the ablation cell, and fitted to the sample injector tube to introduce laser ablation particles to the plasma. The He laser ablation carrier gas serves as the plasma gas and is ionized in the MIP torch. The carrier gas flow rate is controlled by a mass flow controller (Alicat Scientific) nominally set to 30–50 mL/min.

The laser ablation system uses a commercial 266 nm solid state Nd‐YAG laser (New Wave Tempest). The beam optics uses a convergent lens, with the beam being focused onto the sample surface at a 90° angle of incidence. The beam footprint is elliptical with a diameter on the major axis of 100 μm, resulting in fluences on the order of 6 J/cm^2^. This estimate is made given typical non‐gaussian output energies from the laser on the order of 500 μJ, although the beam density would be 12 J/cm^2^ for a gaussian beam profile.

### 1.7 Instrument Control Software and Data Processing

The mass spectrometer described here is controlled using an in‐house LabVIEW‐based software package [57] that enables data collection and storage, as well as digital control of the voltage and QMS RF supplies. Data can be exported as ASCII files that are processed in Excel or MATLAB.

### 1.8 Analytical Sequence Setup

The custom laser ablation cell used in this experimental setup induces a fast washout time of the laser ablation material, with the ion signal appearing ~30–40 ms after initiating ablation and dissipating < 150 ms after ending ablation. Because the commercial laser system used for this study is limited to 10 Hz operations, we originally observed mismatched timing between the emission of the laser pulse (and by extension the generation of ablated particles) and the arrival of analyte ions at the detector. To mitigate this mismatch, we adapted the experiment to employ a scan‐synced laser pulse to produce transient ion signal synced with each mass station/scan segment resulting in a “peak‐hopping” mode of operation. For each isotope mass station in the element menu, a transistor‐transistor logic (TTL) pulse output is initiated prior to the mass scan. This TTL pulse is input into the laser control unit, triggering the laser to fire a single shot. The intensity of the analyte signal at a given mass station is measured directly after each laser shot.

**FIGURE 3:**
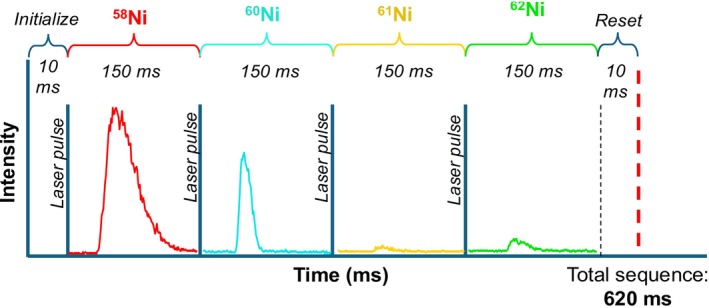
Example of timing diagram of signal acquisition of Ni isotopes from a series of laser pulses, taken from 81 to 19% Ni‐Fe sputtering target. A TTL pulse initiates a single laser shot, with signal acquisition of a specific isotope mass peak scanning across 0.05 amu over a 150 ms dwell time. Note trace contribution to the ^58^Ni signal is from ^58^Fe.

The experimental protocol for each analysis is set up to initially fire a single laser shot, followed by a 10 ms delay. The mass scan begins after the delay, and scans for 150 ms across the peak top (Figure 3), followed by another 10 ms reset period. This timing setup produces 150 ms per peak scan, which is lower than the maximum 10 Hz rep rate supported by the laser system. Each pulse gives a time‐dependent ion signal, with a characteristic peak height, and the area under the peak can be integrated and divided by the number of integration steps. This process reduces shot‐to‐shot signal variability induced by the laser. Scanning across the full mass range requires ~150 ms per peak, producing a 620 ms scan time for four isotope measurements. However, the scan time ultimately depends on the number of peaks in the element menu. Each analysis consists of the collection of 30 s of background data, after which the laser is turned on and the instrument collects 60–90 s of signal data. The measured background signal is later subtracted from the measured ion signal, and the average and standard deviation of the mass trace represent the background‐subtracted analyte ion signal.

## 2 Analytical Methods

Alloys and pure metals were used as samples and standards to validate instrument performance. To demonstrate the capacity to quantify elemental abundances, National Institute of Standards and Technology (NIST) standard reference material (SRM) 1154 stainless steel was used as a bracketing standard, with 316 stainless steel (McMaster Carr, 88 885K77) as the sample. To demonstrate the analysis of isotope ratios, Cu isotopes were measured in pure 99.9% copper (McMaster Carr, 8963K404), and Ni isotopes were measured in a 81–19% Ni‐Fe sputtering target (Kurt J. Lesker, EJTNIFE351A2).

Measurements of each mass peak were made in peak‐hopping mode, with QMS scanning across a small range (0.05 amu) across each peak top. The mass resolution on the QMS was tuned to 1 amu per peak (m/∆m = 63, measured at *m/z* = 63 amu) to maximize signal throughput, and an element menu was setup to scan across each of the isotopes selected for analysis.

## 3 Results

### 3.1 Elemental Quantification via LA‐MIP‐MS

**FIGURE 4:**
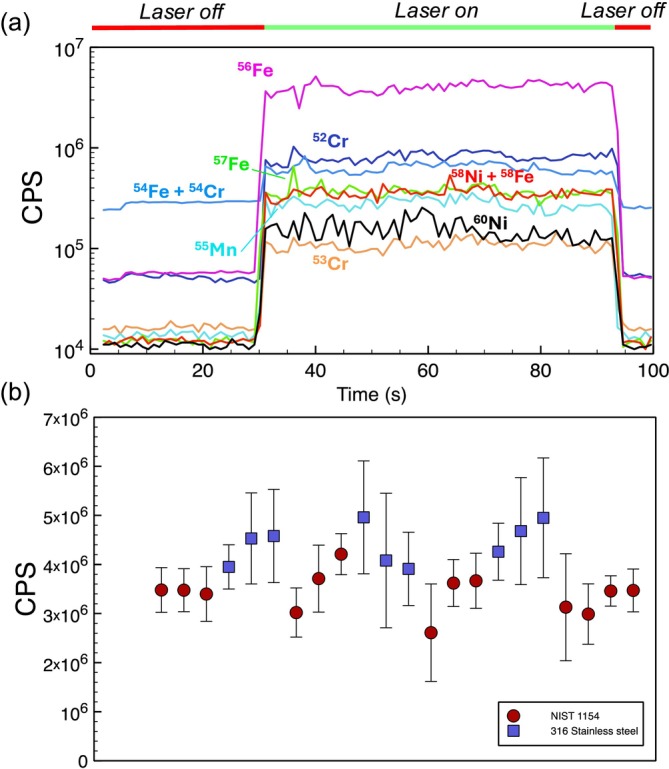
Results of LA‐MIP‐MS measurement of stainless steel. (a) Transient signal trace of sample of 316 stainless steel, with signal data derived from peak intensity. (b) ^56^Fe signal across all analytical runs from NIST SRM 1154 stainless steel and 316 stainless steel sample.

NIST SRM 1154 was used as a reference material and the 316 stainless steel as an unknown, matrix‐matched sample. The analysis included a standard‐sample bracketing sequence, in which 4 sets of SRM 1154 and 3 sets of 316 stainless steel measurements were alternated. Each set of measurements consisted of 3–4 repetitions of the same sample, resulting in 22 distinct measurements (Figure 4b).

Reference concentration values for the 316 stainless steel sample were obtained by XRF analyses (Bruker CTX model) and provided data that could be used for comparative analysis. Elemental abundances of the sample via LA‐MIP‐MS were determined following the quantification protocol outlined by [60] using ^52^Cr as the internal standard.

Figure 4a shows a typical transient signal of laser ablation analysis of 316 stainless steel, in which 30 s of background signal was collected without laser pulsing, followed by ~90 s of laser ablation signal acquisition. The background levels of *m/z =* 52 (5 × 10^4^ counts/s [CPS]), 54 (2 × 10^5^), and 56 (5 × 10^4^) are significantly higher than the background count rates observed in the rest of the isotopes in the element menu (typically ~1 × 10^4^ CPS). Elevated count rates at masses 52, 54, and 56 probably reflect contamination from ^40^Ar^12^C^+^, ^40^Ar^14^N^+^, and ^40^Ar^16^O^+^, respectively, as they are likely derived from N_2_, O_2_, and CO_2_ in the atmosphere. Air leaks in the PTFE tubing used to supply the plasma gas to the instrument and argon leaks through a flow controller are likely culprits for this elevated air and argon background.

Multiple isotope mass stations (e.g., ^54^Fe, ^56^Fe, ^57^Fe, and ^58^Fe as proxies for total Fe) are observed to constrain the abundance of targeted elements and ensure that isobaric interferences did not compromise the data set. The reproducibility of all collected measurements of an element (i.e., all mass stations in all replicate analyses) defines the “external reproducibility” of the abundance of the element. The external reproducibility of the mean signal of ^56^Fe for both the standard and sample, defined by the reproducibility of the measurement on a scan‐by‐scan basis, is seen in Figure 4b. The method of standard‐sample bracketing shows the amount of signal variation between each of the 3 replicate analyses within each analytical set, with the signal varying by ±23% for the SRM and ±35% for the 316 stainless steel sample. Although some triplicate measurements of ^56^Fe show a rising pattern within repetitions of a set of sample measurements, the overall signal stability over time does not show drift over the timescale of the experiment. Further, other isotopes measured in the same set of analyses do not show this similar rising pattern, indicating that the scatter seen in Figure 4b is random and not due to external factors relating to sampling. The variation of the replicate analyses, as shown by ^56^Fe drift ratios between replicate analyses in NIST 1154, was at most 9%, with the drift ratios between analysis sets 3 and 4 only 3%.

**FIGURE 5:**
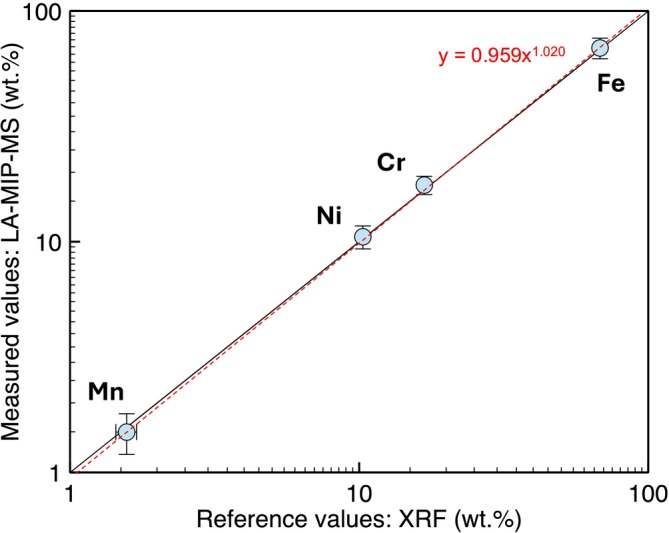
Comparison of the composition of 316 stainless steel as measured by both XRF and LA‐MIP‐MS. Error bars (2σ), taken for XRF values, are smaller than the data symbols. Trendline shows accuracy across two orders of magnitude of concentration.

The accuracy of the LA‐MIP‐MS analyses are compared with the XRF measurements (Figure 5). The accuracy of the inferred abundance of Ni, as quantified independently through ^58^Ni and ^60^Ni, was within 4.1 and 1.4% of ground truth, respectively. The accuracy of Fe was also within 1.3% based on ^56^Fe despite the elevated background. The least abundant isotope in the menu was the least accurate, with ^57^Fe being within 18% of the true values; ^55^Mn, the second‐least abundant, is still accurate to within 5.5%. The 2σ_m_ precision of the quantification analysis, which is defined by the 2σ uncertainty of the external reproducibility divided by the square root of the number of replicate analyses carried out (*n* = 12), ranged from ±9.1 to 22% for all elements (Table 2).

**TABLE 2:** Composition of stainless steel samples.

	316—XRF (wt. %)	316—MIP‐MS (wt. %)	± (wt. %, 2σ_m_)	RSD (%)	Diff (%)
Fe	68.1 ± 0.3	**69.1**	**7.1**	10.3	+1.5
Cr	16.8 ± 0.1	**17.6**	**1.6**	9.1	+4.9
Ni	10.3 ± 0.1	**10.5**	**1.2**	11.3	+1.5
Mn	1.57 ± 0.1	**1.50**	**0.3**	21.9	−4.9

### 3.2 Cu Isotopes

Because the MIP ion source is a low‐power, sub‐atmospheric pressure plasma, the ion temperatures are significantly lower than that of commercial ICP equivalents, which inevitably limit the performance metrics of the former. Fractionation of the analyte can occur both at the ablation site and within the plasma, mainly due to the varying volatilities of the elemental analytes. For example, the temperature at which an element undergoes the phase change from gas to solid, approximated by the 50% condensation temperature (T_50_), influences the composition of laser‐produced aerosols [61, 62, 63]. To examine the effect of volatility in our analyses, we selected Cu as an analyte, as the T_50_ value for Cu (1037 K, [64]) is almost 300 K lower than those of high temperature condensates, including the previously analyzed elements comprising stainless steel.

Cu has only two naturally occurring isotopes: ^63^Cu and ^65^Cu. The Cu isotope ratios were determined for each measurement of a 99.99% pure copper plate after background subtraction (Figure 4a). The precision of each isotope ratio, measured over a 60 s time trace (Figure 4b), ranged from 13 to 30‰ (2σ_m_; *n* ≈ 120 analyses).

**FIGURE 6:**
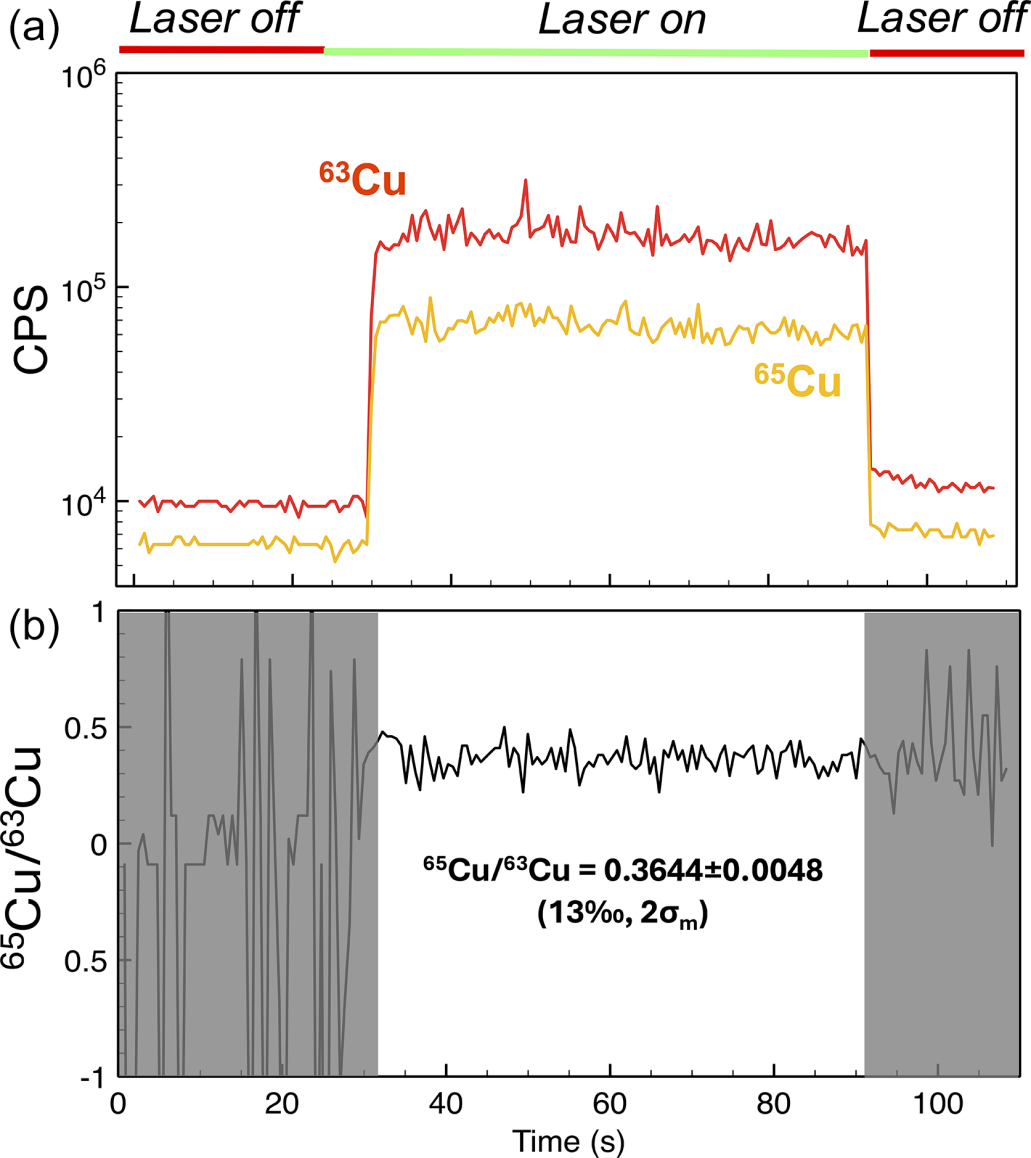
Transient signal trace of Cu taken via LA‐MIP‐MS. (a) Time‐dependent signal trace of both naturally occurring isotopes of Cu, with laser sampling labeled above. Signal data derived from peak integration. Laser ablation was synced to MS pulse output. (b) Background‐subtracted, rolling average (*n* = 5) transient signal traces of the Cu isotope ratio.

Isotope ratios are fractionated relative to their true values due to various factors: laser ablation and plasma plume expansion, mass‐dependent fractionation during sampling in the torch, space‐charge effects, and differences in ion transmission efficiency as a function of mass. No isotope ratio fractionation correction was applied to the reported Cu isotope data, as correction requires at least three isotopes (Equations 1 and 2), while Cu only has two naturally occurring isotopes and no other masses from different isotopic systems (e.g., ^64^Zn) were measured for extrapolation. This lack of fractionation correction likely resulted in the raw measured ratio in Figure 6b to differ from the natural ratio (~0.45) significantly, although measuring this sample relative to a SRM and reporting Cu isotope values in δ notation would alleviate that discrepancy. The external reproducibility of the isotope ratio is evaluated relative to the first measurement, with δ^65^Cu defined as deviation of ^65^Cu/^63^Cu value in parts per thousand (ppt) from the first measured value. Data are plotted in Figure 8a and shows a scatter ranging from +11.1 to −8.3% relative to the reference value.

Given that each experiment lasted only 60 s, and due to the relatively slow duty cycle of the measurements (largely limited by the laser repetition rate), the precision of the measurements presented here represents a lower limit. Higher precision measurements could be supported through longer analysis times, which would increase Poisson counting statistics and reduce the uncertainty on the mean. For example, given the same variance in ^65^Cu/^63^Cu observed in Figure 6b, a 5 min measurement (or 5 × increase in the number of replicate analyses) would reduce the error to 5.9‰ (2σ_m_).

### 3.3 Ni Isotopes

Although Cu represents a moderately volatile element, as the T_50_ value is relatively low compared to other refractory metals, it is important to collect relevant performance data on elements that require high temperature vaporization to facilitate ionization as well. Thus, Ni was also analyzed for isotopic precision and accuracy due to its T_50_ value of 1375 K.

**FIGURE 7:**
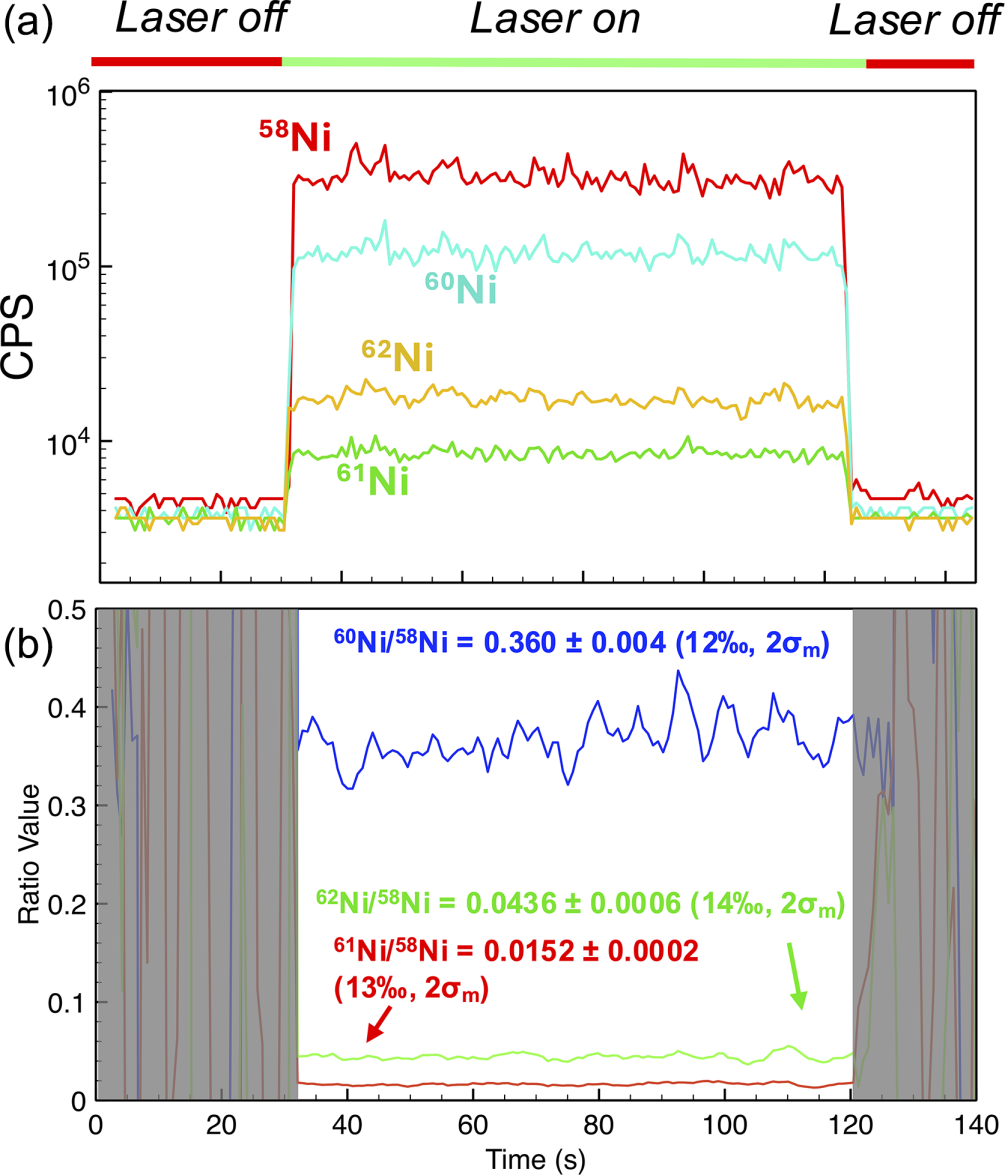
Transient signal trace of Ni taken via LA‐MIP‐MS. (a) Time‐dependent signal trace of four naturally occurring isotopes of Ni, with laser sampling labeled above. Signal data derived from peak integration. As was the case with Cu measurements, laser ablation was synced to MS pulse output. (b) Background‐subtracted, rolling average (*n* = 5) Transient signal traces of Ni isotope ratios.

Ni has five naturally occurring isotopes: ^58^Ni, ^60^Ni, ^61^Ni, ^62^Ni, and ^64^Ni; however, only ^58^Ni, ^60^Ni, ^61^Ni, and ^62^Ni were measured in this study due to significant background at *m/z* = 64. Figure 7 shows a common laser ablation signal for Ni with the laser‐off background being collected for 30 s followed by 60–90 s with laser ablation signal on. The average signal intensity for mass 58 across all 13 scans is 9.1 ± 0.4 × 10^5^ (2σ) CPS, which is a combination of ^58^Ni and ^58^Fe, with only 0.1% of the total signal at mass 58 is contributed from ^58^Fe, which is approximately 10% of the total error and not considered in this analysis. The average background signal at mass 58 is 4.1 ± 5.1 × 10^3^ CPS, giving an average signal to background ratio of 255. Thus, Ni had the highest signal to background ratio of the samples analyzed.

**FIGURE 8:**
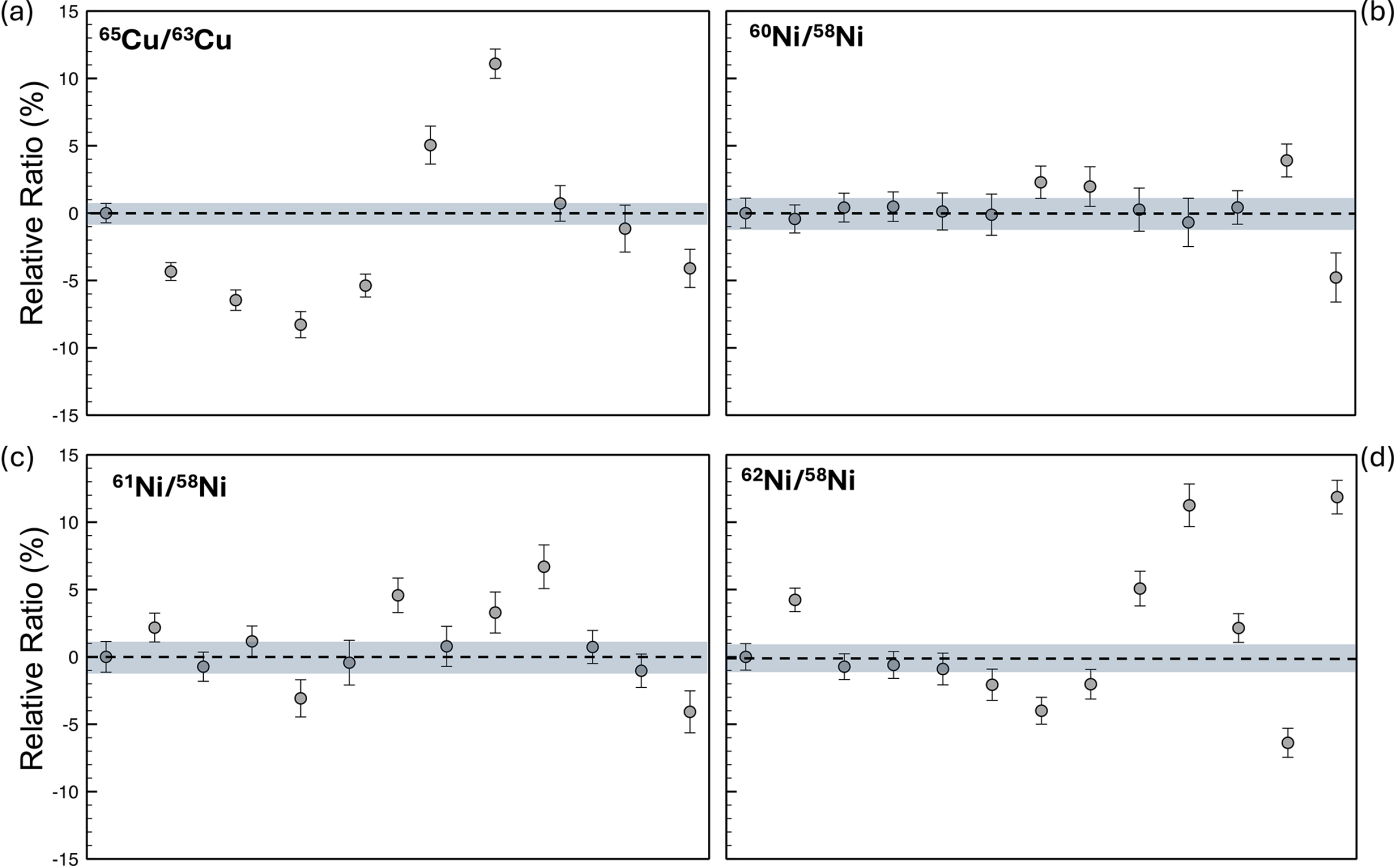
Data scatter and precision of isotope ratios of Cu and Ni. Cu isotopes have not been fractionation corrected, while Ni isotopes have. The three less abundant isotopes of Ni, 60Ni, 61Ni, and 62Ni, were each ratioed to 58Ni. The accuracy was measured relative to the first measurement taken in the sequence, with the grey bar representing the 2σ_m_ precision of the measurement.

To account for mass fractionation, we corrected the measured isotope ratios using a power law, as exponential law corrections are more typically applied to multi‐collector MS systems [65]. The power law is calculated as:
(1)

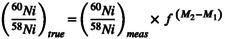




where *f* is calculated as:
(2)

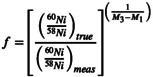




where M_1_, M_2_, and M_3_ are the masses of the three measured isotopes. After fractionation correction, the precision of ^60^Ni/^58^Ni, ^61^Ni/^58^Ni, and ^62^Ni/^58^Ni isotope ratios ranged from ±8.7 to 18‰ (2σ_m_), producing slightly higher uncertainties than the Cu analyses performed previously (primarily due to lower signal‐to‐noise ratios for the low abundance Ni isotopes). To assess external reproducibility, we measured each ratio relative to the first analysis performed, treating the first measurement as a reference value. The external reproducibility of the isotope ratios improved after the fractionation correction was applied, with variances ranging from −5.4 to +5.6% of the initial measurement value (Figure 8b–d).

## 4 Discussion

### 4.1 Sample Detection Efficiency

Detection efficiency is a measure of the number of atoms introduced compared to the number of atoms counted. This factor considers the transport of the analyte (e.g., via laser ablation), atomization and ionization of sample in the plasma, and the efficiency of ion transmission through the mass spectrometer. Detection efficiency varies in commercial ICP‐MS systems as a function of elemental mass, with the lightest elements generally the least efficient at traversing from ion source to detector, and especially due to Columbic repulsion during space charge interactions in the plasma.

Using certified concentration data provided for NIST 1154 and determining the volume ablated per second, one can estimate the atoms of an element introduced per second to the mass spectrometer. The ablated mass of each element or isotope can be compared with the count rate of the mass of interest. For a monoisotopic element like Mn, the signal minus background will identify the ions detected, and this number divided by the number of atoms ablated will give the instrumental detection efficiency for this mass range.

Laser ablation pits are elliptical (typically 100 by 50 μm) and because we are unable to directly measure the pit depth generated by our laser system, we assume a pit depth incision rate of 75–100 nm per shot to perform our calculations for both ablation conditions. Pit depths of 100 nm per shot are typical for UV laser ablation of silicate material with laser energy densities of ~5 J/cm^2^ [66, 67], but because these materials are thermally conductive, as opposed to the semi‐conductive properties of silicates, we assumed a lower ablation efficiency to establish a conservative estimate. Assuming a stainless steel density of 7.9 g/cm^3^, we estimate that an ablation rate of 4 × 10^−9^ g per shot is ablated during laser irradiation. According to the analysis of the major elements in NIST 1154 stainless steel investigated here, the ablated mass translates to ~2 × 10^13^ atoms of ^56^Fe removed per shot. Typical background‐subtracted count rates for ^56^Fe observed in our experiments were on the order of 5 × 10^6^ counts/s, equating to a 2 × 10^−7^ detection efficiency.

Considering the 1 ms integration period used with the pulse counting electronics interfaced to the detector, an idealized sample introduction rate for elemental analytes for NIST 1154 stainless steel would input 5.4 × 10^11^–2.4 × 10^13^ sample ions per second, depending on the analyte abundance. The theoretical maximum signal is calculated using the estimated number of atoms of sample transferred to the instrument from the ablation site, while accounting for the assumed ionization efficiency, laser pulse frequency, and detector time base. The results are reported in Table 3 for each of the isotopes measured. The background‐subtracted signal trace as measured for the elemental analytes in NIST 1154 stainless steel, ranges from 1.2 × 10^5^ to 4.7 × 10^6^ CPS for Cr, Mn, Fe, and Ni. Thus, given the theoretical maximum signal discussed above, the measured signal produces an ion transmission efficiency of 1.7 × 10^−7^ to 8.8 × 10^−7^ for the range of analytes, depending on the analyte.

**TABLE 3:** Measured and calculated instrument figures of merit.

Analyte	Ion signal (CPS)	Theoretical maximum signal (CPS)	Transmission efficiency	Normalized sensitivity (CPS/ppmw)	LOD (ppmw)
^52^Cr^a^	9.4 ± 1.9 × 10^5^	5.7 × 10^12^	1.7 × 10^−7^	7.0	240
^53^Cr	1.2 ± 0.19 × 10^5^	6.3 × 10^11^	1.9 × 10^−7^	7.4	101
^54^Fe^b^	4.6 ± 1.2 × 10^5^	1.6 × 10^12^	2.9 × 10^−7^	10	780
^55^Mn	3.27 ± 0.44 × 10^5^	5.7 × 10^11^	5.7 × 10^−7^	21	33
^56^Fe^c^	4.69 ± 0.68 × 10^6^	2.3 × 10^13^	2.0 × 10^−7^	6.6	240
^57^Fe	4.76 ± 0.67 × 10^5^	5.4 × 10^11^	8.8 × 10^−7^	33	21
^58^Ni^d^	4.89 ± 1.2 × 10^5^	2.4 × 10^12^	2.0 × 10^−7^	7.7	85
^60^Ni	1.87 ± 0.84 × 10^5^	9.1 × 10^11^	2.1 × 10^−7^	7.0	96

^a^ Interference with ArC^+^.

^b^ Interference with ArN^+^, ^54^Cr.

^c^ Interference with ArO^+^.

^d^ Interference with ^58^Fe.

The average transmission efficiency of commercial ICP‐MS has improved over time. Initially, Wälle et al. [68] published detection efficiencies for first‐row transition elements in NIST 610 glass that ranged from 3 × 10^−6^ to 2 × 10^−5^, depending on whether nanosecond or femtosecond laser ablation was used. More recent advances in laser ablation sample transport and ion transmission have brought transmission efficiency to 2–4% for comparable ions of comparable mass ranges [69, 70].

### 4.2 Sample Limit of Detection

The limit of detection (LOD) of an element as analyzed by commercial LA‐ICP‐MS is a function of the sensitivity of the analyte in CPS/ppmw (parts per million by weight, kg/kg), as well as the measured baseline background signal of the system. To calculate the LOD of the first‐row transition elements (FRTE) that comprise the stainless steel sample, we use the following:

(3) $$\mathrm{LOD}=\frac{3\sigma_{m}}{S}$$

where σ_m_ is the standard error of the mean of the background, and S is the normalized sensitivity, calculated in the previous quantification step [60]. Using the measured background and signal count rates determined for each isotope monitored (described above), the measured stainless steel analyte signal, and the calculated concentrations of each analyte in the 316 stainless steel sample, we find the LOD of Cr, Mn, and Ni to range from 33 to 240 ppmw, with the LOD from the various Fe isotopes ranging from 21 to 780 ppmw. This range of calculated LODs shows that quantification of trace elements in stainless steel is possible via the low power, low pressure LA‐MIP‐MS techniques and instrumentation presented here.

The background interference of masses 52, 54, and 56 are likely from argides in the system due to a leak in the Ar ignition gas mass flow controller. While the relative ratios of the argides do not reflect the natural abundances of N, O, and C in the atmosphere, the production of argides is controlled by a number of factors within the plasma. In commercial ICP torches, the ArN^+^ and ArO^+^ abundance varies by 4–6 orders of magnitude depending on temperature (3000–9000 K) and gas flow (0.5–1.1 L/min, [71]). Argide production also varies by instrument configuration depending on the geometry of the 2‐cone interface in commercial ICP interfaces [72], but because our system requires only a single cone, the argide production efficiency is not be comparable to commercial systems. Thus, efforts to reduce the background Ar abundance and a better understanding of the controls on argide production in our specific system will reduce the argide background, thereby lowering the LOD for elements with argide interferences.

#### 4.2.1 Comparison to Heritage Spaceflight Instrumentation

As noted above, previous generations of spaceflight instruments have provided elemental and isotopic data from planetary surfaces. The isotopic composition of volatiles evolved during pyrolysis of Martian materials (e.g., H_2_O, CO_2_, SO_2_, HCl) are regularly measured via the SAM instrument on MSL. Additionally, techniques such as LIBS and APXS have been used for major elemental analysis of basaltic and sedimentary materials and their alteration products on the surface of Mars during, for example, the MER and currently active MSL rover missions. Although major insights into planetary history have been provided by these instruments, the figures of merit (e.g., LOD, uncertainties, Table 4) of each measurement affect which natural processes can be elucidated by the measurements. Here, we discuss the figures of merit of elemental and isotopic analysis produced via several previous spaceflight instruments as compared to LA‐MIP‐MS.

**TABLE 4:** Comparison of spaceflight instrument figures of merit.

Analysis technique	QMS	TLS	LIBS	APXS	LA‐MIP‐MS
Example instrument	SAM (MSL)	SAM (MSL)	ChemCam (MSL)	APXS (MSL)	This work
Isotope ratio Precision (RSD)	±27% 2σ^a^ (for δ^13^C in Martian atmospheric CO_2_)	±8.7% 2σ_m_ ^b^ (for δ^13^C in Martian atmospheric CO_2_)			**±0.8–3% 2σ**_**m**_ **(for δ** ^**60**^ **Ni, δ** ^**61**^ **Ni, and δ** ^**62**^ **Ni in Ni‐Fe alloy)**
Elemental quantification precision, Fe	—	—	±0.78 wt.%^c^	±0.52 wt.%^c^	**±7.1 wt.%**
Elemental quantification accuracy, Fe	—	—	7.54 wt.%^c^	2.02 wt.%^c^	**1.51 wt.%**
Total uncertainty, Fe (2σ RSD)	—	—	±33.6%^c^	±9.1 wt.%^c^	**±10.5%**
Elemental quantification limit of detection	—		Cr: 10’s of ppmw^d^ Mn: 600 ppmw Ni: >1000 ppmw	FeO: 300 ppmw^e^ MnO: 500 ppmw Ni: 50 ppmw Cr: 340 ppmw	**Fe: 21–782 ppmw** **Mn: 33 ppmw** **Ni: 85–96 ppmw** **Cr:101–238 ppmw**

^a^ [5].

^b^ [73].

^c^ [74], data from Marimba‐2 sample.

^d^ [75].

^e^ [76].

### 4.3 Isotope Ratio Measurements

Isotope ratios of several elements in volatile species from Martian sediments and atmosphere have been measured by the SAM instrument onboard the MSL Curiosity rover. SAM employs pyrolysis to measure the composition of volatiles released from sediments at high temperature via QMS and/or SAM's tunable laser spectrometer (TLS) [27, 77]. Because SAM relies on pyrolysis for sample processing, isotope ratios of the lithophile elements in silicates (e.g., Mg, Ca, Ti, etc.) may not be determined using this method. Furthermore, the precision of the measurement of the isotope ratio influences which natural processes can be interpreted from the measured ratio. Processes that induce major isotope fractionation, for example, Rayleigh fractionation due to evaporative volatile loss that drives δ values to ±30‰ or more, can be resolved with error bars of up to ±80% 2σ RSD, or ±24‰ absolute uncertainty.

Multiple analyses of Martian soils and drilled rock powders via SAM have produced isotope ratios documenting large‐scale isotope fractionation, with δ values ranging from −60 to +50 for a number of isotope systems (e.g., C, O, S). For example, SAM QMS provided precise isotope ratio measurements of ±19.6% to 80% 2σ for δ^13^C in evolved CO_2_, ±10.4% 2σ for δ^18^O in evolved CO_2_ [15], and ±59% 2σ for δ^34^S in evolved SO_2_ [78]. SAM also includes a TLS capable of delivering isotope ratio measurements of atmospheric and evolved gases. For example, TLS has reported measurements of atmospheric CO_2_ with uncertainties of ±8.7% (δ^13^C), ±10.4% (δ^18^O), ±20.8% (δ^17^O), and ±28.4% (δ^13^C^18^O) 2σ_m_ RSD [73]. SAM QMS also measured δ^13^C of atmospheric CO_2_, with uncertainties of ±27% [5]. In comparison, the LA‐MIP‐MS prototype described here delivered precision on Cu and Ni isotope ratios ranging from 0.8–3.0% 2σ_m_ RSD, showing the ability for this instrument technique to resolve a larger range of natural processes recorded by isotope ratio fractionation. While the range of uncertainty for volatile elements (e.g., C, N, O, S) is broader compared to that of Ni and Cu due to differences in volatility and extent of natural variation, refractory lithophile isotope measurements have not yet been made in situ via planetary mass spectrometry before. Thus this comparison is necessary for lack of other such examples.

### 4.4 Elemental Detection Limits

In addition to isotope ratios, LIBS and APXS have provided elemental abundances of Martian sediments. The LIBS ChemCam instrument on MSL reports LODs on Mars ranging from 0.3 to 25 ppmw for Li, and 46 to 973 ppmw for Ba [79], reflecting the dependence of sensitivity of LIBS measurements to standoff distances, spectral interferences, etc. The LA‐MIP‐MS prototype instrument provides similar performance metrics, albeit with lower LODs reported for several first row transition elements. For example, ChemCam's reported LODs for Mn and Ni are > 460 ppmw and > 1000 ppmw, respectively [75]; the LODs for Mn and Ni reported here improve on those values by more than 1–2 orders of magnitude.

For APXS, LODs depend on the target element, sample matrix, and experimental conditions [24]; extended observations (i.e., integration times) are often required to build the statistics to precisely quantify elemental abundances. With an ideal instrument geometry, APXS LODs of 340 ppmw, 380 ppmw, and 50 ppmw were reported for Cr, Mn, and Ni, respectively [76]. However, limiting integration times compromises these figures; for example, the detection limit for Ni increases by a factor of > 7× (from 50 to 360 ppmw) in a “touch and go” scenario consisting of 20 min of integration time, versus an overnight analysis [26]. The LA‐MIP‐MS prototype described here improves on the LODs for Cr and Mn regardless of the integration time, and approaches that for an overnight Ni measurement. The LOD of LIBS and APXS also enables further trace element quantification, with Li, Ba, Rb, and Sr. being quantified via LIBS with abundances from 10’s to 100’s of ppmw, and Co, Ni, Cu, Zn, Ga, Ge, and Br showing LODs via APXS to as low as 30 ± 3 ppmw [26].

The data discussed above suggest that the LA‐MIP‐MS instrument may deliver LOD value that are an order of magnitude or more improvement on trace element quantification in solid material, compared to LIBS and APXS. Additionally, this method requires significantly less integration time (~90 s per transient analysis) and fewer calibration points (~10–15) than LIBS or APXS. The previously deployed instrumentation requires 6–8 h of integration to achieve these LODs for APXS, with > 300 calibration points for LIBS.

### 4.5 Precision and Accuracy of Elemental Quantification

For both APXS and LIBS, the precision and accuracy of the measurements define the total uncertainty. Analytical precision can be higher with these two techniques, where measurement accuracy is often lower. For example, the 2σ uncertainty of FeO measurements of the Marimba‐2 mudstone on Mars, which had a FeO content of 22.5 wt.%, can be broken down for each technique, yielding an accuracy of 2.02 wt.% and precision of ±0.52 wt.%, for a total absolute uncertainty of ±2.04 wt.% 2σ for APXS (±9.1% 2σ RSD), and an accuracy of 7.54 wt.% and precision of ±0.78 wt.% for a total absolute uncertainty of 7.58 wt.% 2σ for LIBS (±33.6% 2σ RSD) ([80, 81, 74]). During the quantification of Fe in stainless steel, our LA‐MIP‐MS instrument yielded an accuracy of 1.51 wt.% and precision of ±7.1 wt.% 2σ_m_, for a total absolute uncertainty of ±7.26 wt.% (±10.5% 2σ_m_ RSD).

These results suggest that our LA‐MIP‐MS technique may provide instrument uncertainties lower than LIBS and approaches the uncertainties of APXS. As discussed above with LOD, the uncertainties of APXS are also dependent on integration time, where an overnight analysis would provide higher precision and accuracy than the “touch and go” analysis. In contrast, the LA‐MIP‐MS quantification produced here is based on a series of ~90s transient signal analyses, which means that quantification can be carried out in minutes. Thus, the science output per unit of time of LA‐MIP‐MS could be much greater than APXS. It should be noted that the number of repeat measurements (e.g., *n* = 9 used in this work) reduces the 2σ_m_ uncertainty. If 2σ uncertainties are applied to the quantification, which range from ±16% for Cr to ±25% for Mn (RSD), the uncertainties here would still be smaller than those produced for LIBS and like those of APXS. Thus, with more frequent sampling, the uncertainty can decrease even further.

Previous work has shown the ability to produce meaningful scientific insights with a wide range of measurement uncertainty. For example, Treiman et al. [74] showed that major element analysis (e.g., SiO_2_, FeO, MgO, Al_2_O_3_, CaO, K_2_O, Na_2_O, TiO_2_) of both LIBS and APXS can enable various basalt classification schemes at the 2σ level, although LIBS can be more ambiguous than APXS due to greater uncertainty in major element analyses. An analysis of a surficial basalt carried out by LIBS produced a 2σ RSD ranging from ±20% (SiO_2_) to ±81% (MgO, [82]), while APXS delivered lower uncertainties of ±5.9% (SiO_2_) to ±23% (MgO) RSD on the same sample, respectively [80, 81]. Other LIBS and APXS measurements of major elements have documented the diversity of crustal igneous rocks and determined the provenance of multiple sources of basalt in Gale crater with uncertainties as high as ±80% 2σ for some major element analyses with LIBS [75]. Given the LODs and uncertainties reported here, LA‐MIP‐MS may be able to deliver additional insights into geologic processes in planetary environments than previously thought. The additional context of lithophile element isotope ratios further sheds light on a number of natural processes previously inaccessible to state‐of‐the‐art spaceflight analytical techniques.

#### 4.5.1 Considerations for Mission Operation and Deployment

The instrument described here is designed for future deployment on planetary surfaces for in situ geochemical analysis. While the overall architecture of the instrument and its performance metrics show the feasibility of this technique to meet planetary science objectives, further considerations are needed for future mission deployment.

The figures of merit for elemental and isotopic analysis discussed here have been collected using thermally conductive metallic material, while the science goals of a planetary mission would likely entail LA‐MIP‐MS analysis of silicate, carbonate, sulfate, or other geological phases. Because every unique planetary material is characterized by a unique UV absorptivity, electrical conductivity, and thermal inertia (to name a few), photon‐substrate coupling between the incident laser light and the target will inherently vary, leading to variations in plume dynamics, particle size distributions, etc. Because the MIP ion source operates at a lower temperature than a conventional high‐power ICP torch, larger particle size distributions will inevitably be atomized at a lower efficiency. Incomplete atomization of large ablation‐derived particles can be mitigated using a femtosecond laser, which has been shown to reduce the average particle size distribution, producing more stoichiometrically representative ablation material and enabling more efficient atomization of material in the torch. However, as of this writing, no such laser exists as a spaceflight instrument. Nanosecond lasers offering deeper UV wavelengths (e.g., 213 nm) could improve photon‐substrate coupling with silicates compared to the 266 nm sources baselined on the ExoMars Mars Organic Molecule Analyzer (MOMA) and Dragonfly Mass Spectrometer (DraMS) instruments.

Deployment of a LA‐MIP‐MS for spaceflight would require a reservoir of He gas, which represents a consumable and could limit mission lifetime. However, He reservoirs have been flown on a number of historical planetary missions. For example, two He reservoirs were flown as part of the MSL/SAM instrument, both characterized by a volume of 180 cm^3^ and filled to a pressure of 140 bar at room temperature [27]; per the ideal gas law, this equates to 25 L of He per reservoir at atmospheric pressure. If three reservoirs were flown, and the LA‐MIP‐MS uses a flow rate of 50 SCCM, that would deliver 25 h of continuous gas flow for the experiment. Given that 16 replicates (3x SRM, 10x samples, then 3x more SRM) of 90 s duration signal measurements require less than 30 min to carry out, this gas load would enable up to > 50 end‐to‐end experiments during a mission. The number of full analyses could increase if fewer than 10 replicate sample measurements are performed per analysis.

While the instrument discussed here is constructed mainly of commercial hardware, the current best estimate of a spaceflight version of the LA‐MIP‐MS is estimated to be < 16 kg total. Previous versions of spaceflight QMS assemblies typically weigh 5–7 kg, with 5 kg mass for flight electronics and 2–3 kg for the laser. The QMS requires only 40–50 W of peak power, while laser emission requires 10 W and the RF and DC power supplies require 2–6 W. Accounting for the 20–30 W of the plasma ion source, LA‐MIP‐MS requires a total of < 100 W for surface operations. This power use is comparable to previous spaceflight instrument packages, e.g. MOMA (11.5 kg, 133 W max power, [52, 87]), but more demanding than spectroscopy‐based techniques such as LIBS (10.6 kg, 64.5 W, [21]) and APXS (0.57 kg, 1.3 W, [83]).

The precision, accuracy, and LOD of this instrument is limited by a number of factors, and specifically, the mass analyzer selected to carry out these analyses can influence performance metrics as well. In commercial ICP‐MS systems, the precision of a QMS‐based instrument is limited to ±0.1‰ (2σ) for most lithophile isotope ratio measurements. However, the application of magnetic sector and multi‐collector ICP‐MS instruments improves signal stability dramatically, reducing uncertainties to the parts‐per‐million level in some isotope systems. While this effort integrated a MIP ion source to a QMS, future work can interface this plasma ion source to other mass spectrometer configurations as well. Magnetic sector instruments, which are known for signal stability, have been flown in multiple previous missions, including for the Apollo 15 and 16 [2], Viking [14], and Rosetta [84] missions. Instrument concepts have already been developed to interface a low‐power plasma to a sector field instrument [85], potentially enabling geochronology or other higher precision isotope ratio measurements. Other mass spectrometer types, such as time‐of‐flight [51, 56, 86] or Orbitrap [54, 55] are being developed for spaceflight and can be fitted with a MIP ion source for enhanced instrument performance as well.

### 4.6 Overall Performance of LA‐MIP‐MS

The results presented here include the quantification of elemental abundances and isotope ratios of solid material by LA‐MIP‐MS, with uncertainties ranging from ±9.1–22% 2σ_m_ RSD for major element quantification, and ±0.87–3.0% 2σ_m_ for isotopic ratios. The LODs calculated in Table 3 are higher than those estimated for APXS in some cases, but elemental quantification using isotopes with LODs > 100 ppmw occurs on peaks with argide interferences. Thus, efforts to reduce argon background in the gas delivery system may lower these LODs, improving instrument performance for these isotopes. Importantly, previous efforts to measure elemental and isotopic chemistry in situ on planetary surfaces required multiple instruments working in tandem, with elemental quantification carried out using LIBS or APXS and isotope ratios of the volatile elements being determined via SAM. This work demonstrates the ability to provide all necessary measurements with a single instrument technique, further adding to the capabilities of the state of the art for in situ geochemistry of planetary surfaces.

## 5 Conclusions

We have demonstrated the performance of a LA‐MIP‐MS using a low power (30 W), low gas flow (≤50 mL/min) microwave plasma as the ion source. Our experiments were carried out using a series of metallic samples (i.e., 316 stainless steel, NIST SRM 1154, pure Cu, and an Fe‐Ni alloy) and sampling them by laser ablation to demonstrate the feasibility of this technique. The working prototype characterized here is currently capable of measuring first row transition elements with detection limits down to ~21 ppmw. Our isotopic analyses produced external reproducibility within ±12% of a standard value, with ratio precision ranging from 0.8 to 3% for Cu and Ni isotopes. Although this effort documents a unique application of laser ablation sample introduction coupled to sub‐atmospheric pressure microwave plasma source, more work is needed to enhance sensitivity and characterize the instrument's capability for analyzing planetary analog samples, such as silicate materials (e.g., NIST 610, 612 glass, USGS standard basalts). However, we have demonstrated the feasibility of this technique while using orders of magnitude less power and gas consumption than commercial systems. LA‐MIP‐MS can be considered as a spaceflight instrument for in situ chemical analysis of geologic material on planetary surfaces.

## Author Contributions

**Benjamin J. Farcy:** funding acquisition, investigation, conceptualization, writing – original draft, methodology, formal analysis. **Jacob Graham:** investigation, writing – review and editing, validation, methodology. **Madeline Raith:** formal analysis, investigation, validation, writing – review and editing. **Ricardo Arevalo Jr.:** conceptualization, investigation, funding acquisition, writing – review and editing, supervision. **Mazdak Taghioskoui:** investigation, funding acquisition, writing – review and editing. **Sierra Budinoff:** conceptualization, visualization. **Amy McAdam:** investigation, funding acquisition, writing – review and editing. **Jane Lee:** investigation, funding acquisition, writing – review and editing. **Ryan M. Danell:** investigation, visualization, writing – review and editing, software. **Desmond A. Kaplan:** investigation, visualization, writing – review and editing, software. **Cynthia Gundersen:** conceptualization, investigation, funding acquisition, visualization. **William F. McDonough:** conceptualization, investigation, funding acquisition, writing – review and editing, supervision.

## Supporting information


**Data S1:** Supporting information.

**Data S2:** Supporting information.

## Data Availability

The data that supports the findings of this study are available in the supplementary material of this article.
